# Engineered Bacteria for Short-Chain-Fatty-Acid-Repressed Expression of Biotherapeutic Molecules

**DOI:** 10.1128/spectrum.00049-23

**Published:** 2023-03-20

**Authors:** Kineret Serebrinsky-Duek, Maria Barra, Tal Danino, Daniel Garrido

**Affiliations:** a Department of Chemical and Bioprocess Engineering, School of Engineering, Pontificia Universidad Católica de Chile, Santiago, Chile; b Department of Biomedical Engineering, Columbia University, New York, New York, USA; The Pennsylvania State University

**Keywords:** biosensor, biotherapeutics, genetic circuit, GM-CSF, propionate, butyrate

## Abstract

Short-chain fatty acids (SCFA) such as propionate and butyrate are critical metabolites produced by the gut microbiota. Microbiome dysbiosis resulting in altered SCFA profiles is associated with certain diseases, including inflammatory bowel diseases (IBD), characterized by a reduction in butyrate concentration and active intestinal inflammation. There is an increasing interest in the use of engineered bacteria as diagnostic and therapeutic tools for gut diseases. In this study, we developed genetic circuits capable of sensing SCFA concentrations to build biosensors that express a response protein (superfolder green fluorescent protein [sfGFP]) in amounts inversely proportional to the SCFA concentration. We also built biotherapeutics expressing the cytokine granulocyte-macrophage colony-stimulating factor (GM-CSF) using the same logic. The propionate biotherapeutic expressed larger amounts of mouse GM-CSF in the absence of propionate. The butyrate biotherapeutics presented the expected behavior only at the beginning of the kinetics and an accelerated response in the absence of butyrate. Overall, these genetic systems may function as complementary diagnostic tools for measuring SCFAs and as delivery vehicles for biotherapeutic molecules.

**IMPORTANCE** Short-chain fatty acids are key molecules produced by the gut microbiome. Their concentrations are altered in certain diseases. Here, we created molecular biosensors that quantify the absence of propionate and butyrate, using logic “NOT” gates and bacterial promoters. Finally, we show that these genetic systems could be useful for the delivery of therapeutic molecules in the gut, in the absence of these acids.

## INTRODUCTION

Inflammatory bowel diseases (IBD) primarily comprise Crohn’s disease and ulcerative colitis. These chronic, multifactorial, and recurrent diseases are characterized by severe inflammation in the gastrointestinal tract and an exacerbated immune response to the intestinal microbiota (1, 2). IBD currently has no cure, and treatments frequently target inflammation and symptom management but not the cause (1, 3, 4). It is estimated that more than 6.8 million people worldwide are living with this type of disease as of 2019 (5), and its prevalence has been increasing in recent years (6). Although the causes are not fully understood, they are known to be a complex combination of immunological factors, environmental factors, genetic predisposition, and dysbiosis of the intestinal microbiota, among others (1–3, 7). Microbial dysbiosis is defined as a harmful alteration of the composition of the organisms that live in the gut, which, among other effects, alters the abundance of metabolites that these organisms provide to the host (2, 8).

Short-chain fatty acids (SCFA) are among the most relevant metabolites of intestinal function produced by gut microbiota. SCFA are primarily produced by anaerobic bacterial fermentation of nondigestible fibers (9, 10). The most relevant SCFA are acetate, propionate, and butyrate, which are typically found in a 3:1:1 ratio and reach concentrations of 50 to 150 mM in the human colon (9, 11, 12). These metabolites are the primary energy sources for colonocytes, have been shown to maintain intestinal homeostasis because of their anti-inflammatory and protective effects on the intestinal epithelium, and participate in the regulation of multiple cellular processes (9, 11, 13–15). Lower concentrations of SCFA have been observed in patients with IBD (9). Studies analyzing the phylum and species gut composition have shown a decrease in propionate-producing and butyrate-producing bacteria in the mucosa and stool of patients with IBD (7, 16–18). In addition, a decrease in these metabolites has been observed under active inflammatory conditions (19, 20). Although it is still unclear whether intestinal dysbiosis is a cause or consequence of IBD (21), these and other studies suggest a correlation between decreased propionate and/or butyrate concentrations and active intestinal inflammation conditions.

There is an increasing interest in the use of engineered bacteria as diagnostic and therapeutic tools for different diseases, such as IBD. Many examples of these genetically modified organisms have been tested preclinically or clinically (22, 23). A biosensor is a microorganism designed to detect one or more specific signals, as biomarkers, under a desired condition, generating a quantifiable response (23). Bacterial biosensors can detect substances that may not be quantifiable by other methods or may not be quantifiable because signals can be unstable or because they may be present specifically in the disease environment (22). In addition, it is possible to design genetic circuits with different conditions and logic to sense more than one signal and increase the specificity of diagnosing a particular disorder. Therefore, biosensors have the potential to act as a complementary, noninvasive, and precise diagnostic tool. Similarly, a live biotherapeutic corresponds to a microorganism that functions as a delivery vehicle carrying a particular therapeutic substance to a specific part of the body, producing it only where it is required (22, 23). In this way, systemic drug exposure is reduced and target specificity is improved, reducing side effects and/or promoting the efficacy of treatment (22, 24). Biosensors and live biotherapeutics can be combined to create sensing and response systems (23). As a result, a therapeutic substance can be produced exclusively in the presence of a biomarker, adding specificity and regulation to the engineered live biotherapeutic bacteria.

Reduced SCFA production in the gut has been associated with IBD and other inflammatory diseases. Therefore, sensing and releasing biotherapeutics in the presence of low SCFA concentrations might be of interest. In this study, we designed and constructed genetic circuits that can sense low propionate or butyrate concentrations and express a protein of interest inversely to the concentration. We developed and characterized three biosensors with these circuits that can produce increasing amounts of superfolder green fluorescent protein (sfGFP) in the presence of low propionate or butyrate concentrations. We also developed three live biotherapeutic sensors that can sense decreased propionate or butyrate concentrations so that they can express a cytokine, mouse granulocyte macrophage-colony-stimulating factor (mGM-CSF), that may counteract inflammation.

## RESULTS

### Inducible promoter selection.

The propionate-responsive system developed in this study was based on the previously constructed pPro24 plasmid (25). This plasmid is based on the *prpBCDE* operon of E. coli, facilitating propionate catabolism. The operon promoter, P*_prpB_*, is regulated by the transcriptional activator PrpR in the presence of propionate. The plasmid pPro24, which was shown to induce differential expression in response to different propionate concentrations (25), contains the *prpR* gene transcribed by its own promoter and GFPuv, the UV-excitable green fluorescent protein which is regulated by P*_prpB_*, both of which are transcriptional units used in our system.

For a butyrate-responsive system, we first evaluated whether the butyrate-induced promoter candidate, the P*_pchA_* promoter (26), induced expression of a gene downstream it according to butyrate concentrations. P*_pchA_* is the promoter of the *perC* homologue A (PchA) regulator, one of the Pch regulators responsible for activating transcription of the locus of enterocyte effacement 1 (LEE1) operon in enterohemorrhagic Escherichia coli (EHEC) (26, 27). This operon contains several structural genes and auxiliary proteins necessary to form the type III secretion machinery of the LEE pathogenicity island in enteropathogenic and enterohemorrhagic E. coli (27). The induction of LEE1 genes by butyrate is directly mediated by the PchA protein and the gene promoter plays a key role in this regulation (26).

**FIG 1:**
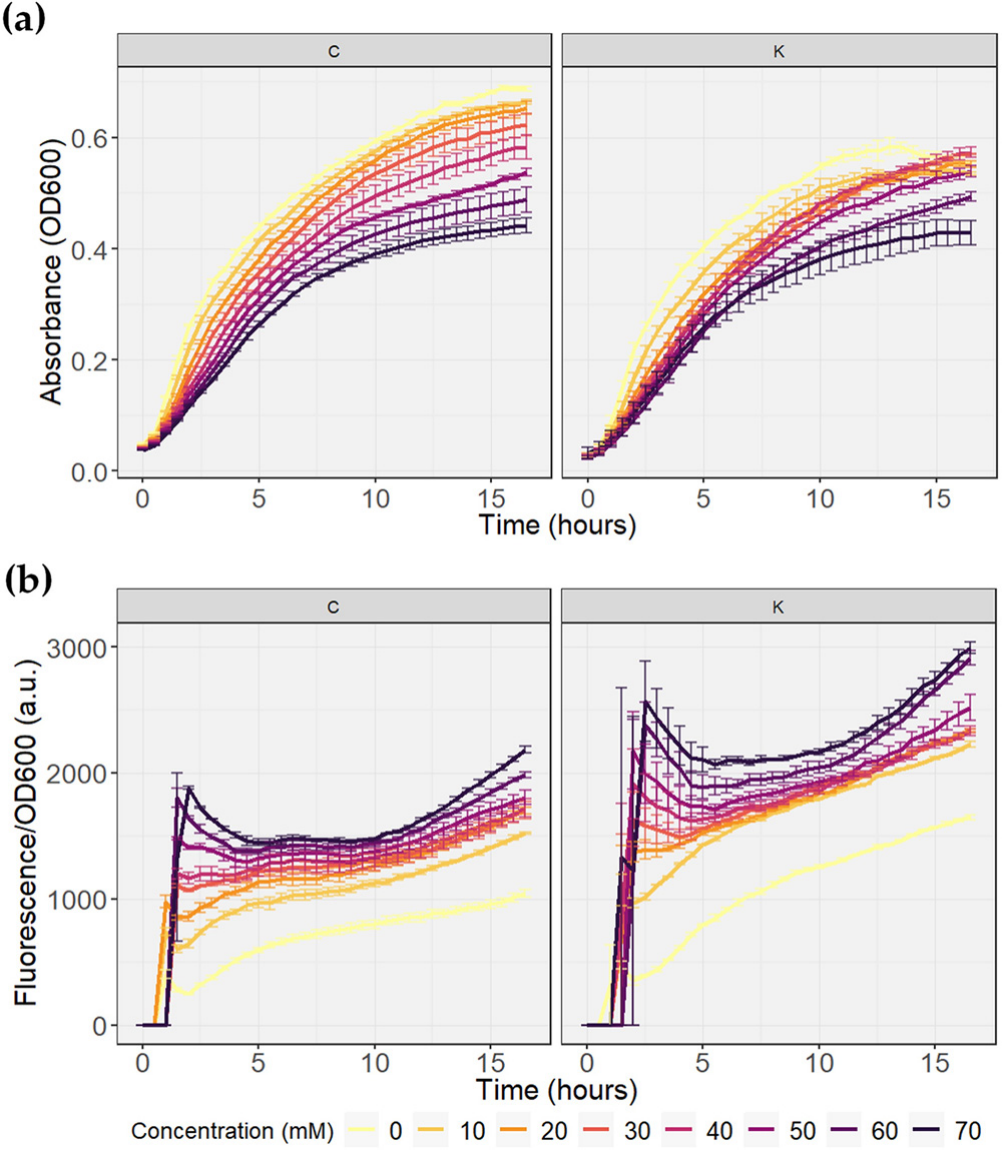
Growth kinetics of BL21 bacteria transformed with the plasmids PpchA_*sfGFP_*C (C) and PpchA_*sfGFP_*K (K) to evaluate the dynamics of P*_pchA_* induction in vectors with different antibiotic resistances. The assay was performed in duplicate. (a) Growth curves. (b) Fluorescence normalized to growth. a.u., arbitrary units.

Two promoter test vectors with different resistances to antibiotic genes were assembled to evaluate whether the P*_pchA_* promoter could induce differential expression in response to different butyrate concentrations. The growth and normalized fluorescence curves of the bacteria transformed with the promoter test vectors are shown in Fig. 1. Bacterial growth was affected by the concentration of butyrate in both plasmids, as has been seen in other E. coli strains (10, 26) (Fig. 1a). The higher the concentration of the compound, the lower the growth rate. This effect was more evident in bacteria carrying the PpchA_*sfGFP_*C plasmid (Table 1), where even a negative correlation was observed between the measured absorbance and butyrate concentration. Growth at lower butyrate concentrations was also higher in bacteria with the PpchA_*sfGFP_*C plasmid than with the PpchA_*sfGFP_*K plasmid (Fig. 1a). In contrast, the fluorescence normalized with both vectors exhibited concentration-dependent behavior (Fig. 1b). As the concentration of butyrate in the medium increased, higher expression of sfGFP was induced in the bacteria. From this, it can be concluded that the activity of the P*_pchA_* promoter does have an induction that can be regulated by the butyrate concentration in the E. coli BL21 strain and allows the induction of protein expression in amounts proportional to the concentration.

**TABLE 1:** Plasmids used in this study

Plasmid	Antibiotic	Description	Use
pPro24	Carbenicillin	High-copy-number plasmid with GFPuv regulated by P*_prpB_*, and PrpR; purchased from Addgene	Backbone
Ptac_*sfGFP_*ColE1_C	Carbenicillin	High-copy-number plasmid; kindly donated by Tal Danino’s laboratory at Columbia University	Backbone
Ptac_*sfGFP_*ColE1_K	Kanamycin	High-copy-number plasmid; kindly donated by Tal Danino’s laboratory	Backbone
pPro_*mGMCSF*	Carbenicillin	Plasmid containing the mGM-CSF gene sequence	Template
Ptac_*sfGFP_*SC101	Chloramphenicol	Low-copy-number reporter plasmid; kindly donated by Tal Danino’s laboratory	Response plasmid
PprpB*_lac*I	Carbenicillin	*lacI* cloned downstream of the P*_prpB_* promoter in pPro24	Propionate sensor
PpchA_*sfGFP_*C	Carbenicillin	P*_pchA_* promoter cloned upstream of the sfGFP gene in Ptac_*sfGFP_*ColE1_C	Promoter test
PpchA_*sfGFP_*K	Kanamycin	P*_pchA_* promoter cloned upstream of the sfGFP gene in Ptac_*sfGFP_*ColE1_K	Promoter test
PpchA_*lacI*_C	Carbenicillin	*lacI* cloned downstream of the P*_pchA_* promoter in PpchA_*sfGFP_*C	Butyrate sensor
PpchA_*lacI*_K	Kanamycin	*lacI* cloned downstream of the P*_pchA_* promoter in PpchA_*sfGFP_*K	Butyrate sensor
Ptac_*mGMCSF*_SC101	Chloramphenicol	mGM-CSF gene cloned downstream of the P*_tac_* promoter in Ptac_*sfGFP_*SC101	Response plasmid

It was also observed that bacteria carrying the PpchA_*sfGFP_*K plasmid (Table 1) reached a higher level of normalized fluorescence at each concentration used (Fig. 1b). Furthermore, when calculating the difference in normalized fluorescence levels between 0 and 70 mM butyrate, there was a greater difference expressed with the PpchA_*sfGFP_*K plasmid than with the PpchA_*sfGFP_*C plasmid for each hour from 2 h of kinetics.

Finally, it is important to note that the P*_pchA_* promoter had a high basal transcription rate, as seen in the butyrate-free curve in both vectors (Fig. 1b). The expression and accumulation of sfGFP without butyrate reached 50% of the normalized fluorescence achieved with the highest concentration of butyrate used, in both cases.

### Circuit design.

**FIG 2:**
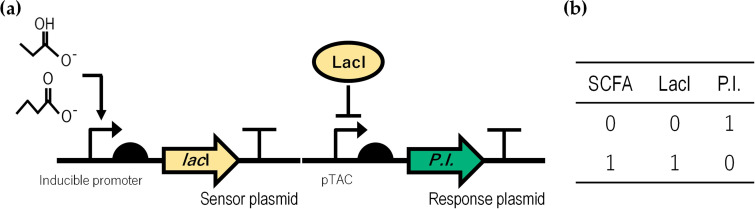
The two-plasmid system developed in this study. P.I., protein of interest. (a) Gene circuit with a NOT logic. Each transcriptional unit represents a plasmid in the system proposed in this study. (b) Truth table of the system logic. 0 represents absence and 1 represents presence.

Since both promoters chosen were activated with propionate or butyrate, it was necessary to invert the input signal of the system to achieve minimal to no expression of the response protein at high concentrations of the SCFA and, conversely, maximum expression at low metabolite concentrations. The gene circuits shown in Fig. 2a were designed for this purpose. The system behavior corresponds to a “NOT” logic gate, and the circuit truth table is shown in Fig. 2b. The system developed in this study requires two plasmids, each one with a transcriptional unit in the circuit. The sensor plasmid had a high-copy-number replication origin site (ColE1), and the response plasmid had a low-copy-number replication origin site (SC101) in order to ensure the plasmids’ compatibility, ensure sufficient expression of the repressor to control the P*_tac_* promoter, and minimize the P*_tac_* promoter leakiness effects. In the sensor plasmid, the inducible promoter is activated by the corresponding SCFA and induces LacI protein expression. LacI, in turn, represses the P*_tac_* promoter, which regulates the expression of the protein of interest: sfGFP for the biosensors and mGM-CSF for the biotherapeutics on the response plasmid. Thus, when propionate or butyrate is present, LacI is expressed, which represses the expression of the protein of interest, and when the SCFA is absent, LacI is not expressed, allowing the expression of the protein, generating the NOT logic. As both promoters have concentration-regulated induction of their inducers, LacI expression is also expected to be proportional to the concentration of the compound. Consequently, it is expected that the expression of sfGFP or mGM-CSF will be inversely proportional to the propionate or butyrate concentration used.

### Biosensor development.

**FIG 3:**
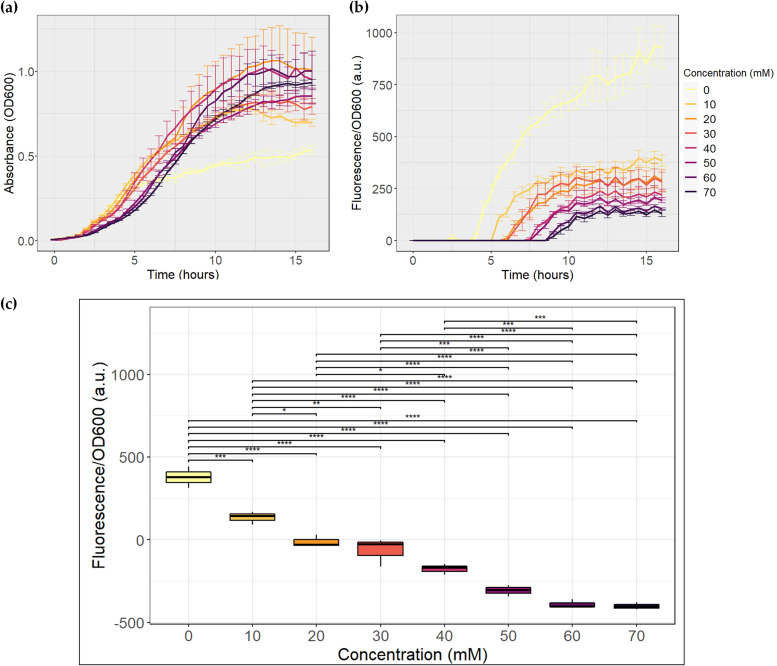
Evaluation of the propionate biosensor. The assay was performed in triplicate. a.u.: arbitrary units. (a) Growth curves. (b) Fluorescence normalized to growth. (c) Results of *post hoc* analysis with Tukey’s test for the propionate biosensor at 6 h of growth. *, *P* ≤ 0.05; **, *P* ≤ 0.01; ***, *P* ≤ 0.001; ****, *P* ≤ 0.0001.

**FIG 4:**
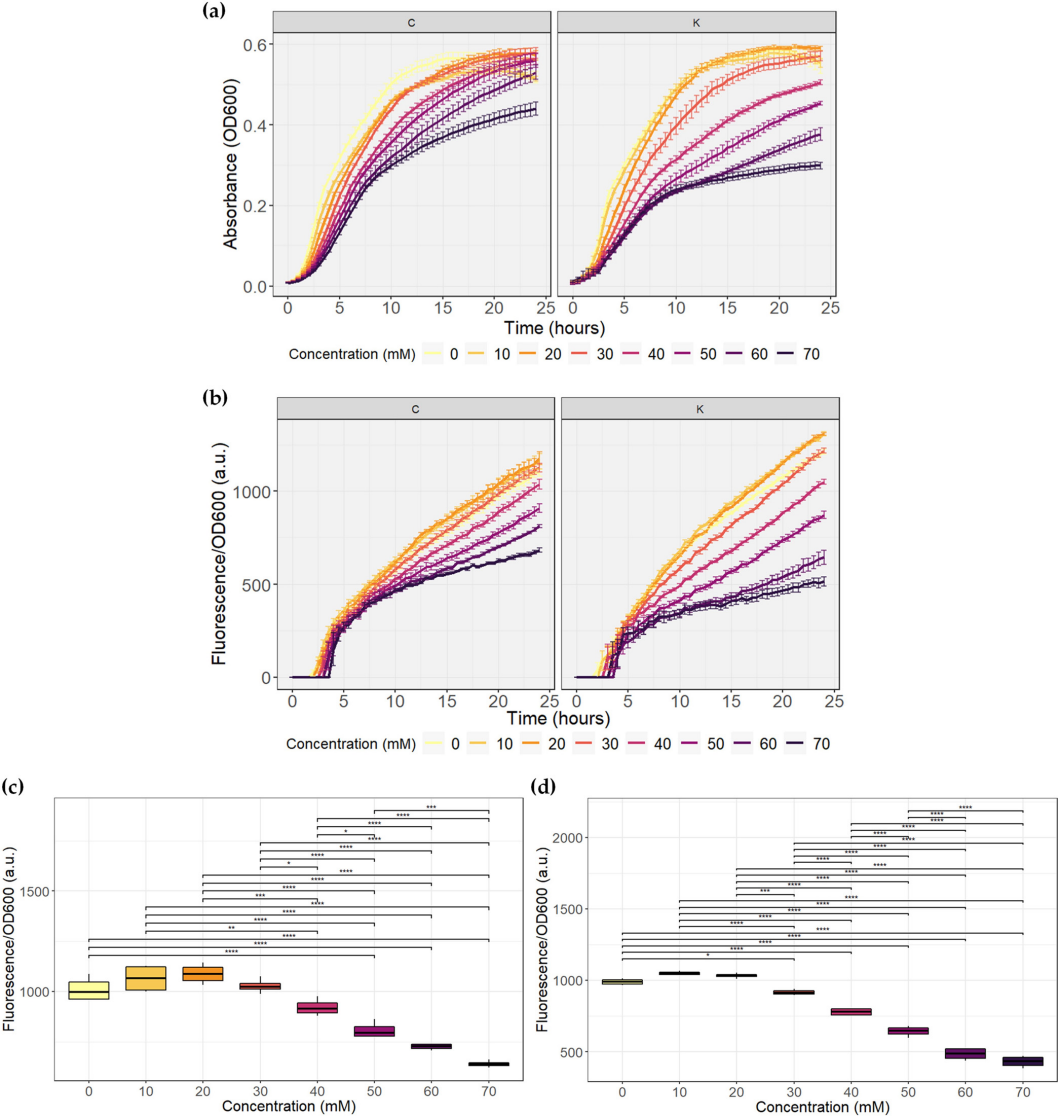
Evaluation of butyrate biosensors C and K. The assay was performed in quadruplicate. a.u., arbitrary units. (a) Growth curves. (b) Fluorescence normalized to growth. (c) Results of *post hoc* analysis with Tukey’s test for butyrate biosensor C at 21 h of growth. (d) Results of *post hoc* analysis with Tukey’s test for butyrate biosensor K at 17.5 h of growth. *, *P* ≤ 0.05; **, *P* ≤ 0.01; ***, *P* ≤ 0.001; ****, *P* ≤ 0.0001.

The propionate biosensor was created by cotransforming E. coli DH5α with the propionate sensor plasmid PprpB*_lac*I and the reporter plasmid Ptac_*sfGFP_*SC101 (Table 1). Butyrate biosensors were created by cotransforming E. coli BL21 with the same reporter plasmid and the butyrate sensor plasmid PpchA_*lacI*_C or PpchA_*lacI*_K (Table 1), referred to as butyrate biosensor C and butyrate biosensor K, respectively. The kinetics were evaluated until the systems reached the stationary growth phase at all concentrations (Fig. 3 and 4).

**(i) Propionate biosensor.** To ensure adequate bacterial growth and the use of propionate to activate the genetic circuit, we grew our strain in LB (Luria-Bertani) medium supplemented with propionate at each concentration in triplicate (Fig. 3). The bacteria grew in the presence of every concentration of propionate used, but the optical density at 600 nm (OD_600_) achieved by the cultures with propionate was higher than that in the absence of the compound (Fig. 3a). The duration of the lag phase generally increased with propionate concentration, indicating that the bacteria took longer to adapt to the medium when it was enriched with propionate.

An inversely proportional relationship between propionate concentration and fluorescence was observed, in accordance with our objective (Fig. 3b). The amount of time required for fluorescence to be higher than the medium fluorescence increased considerably with propionate concentration, which appears to be related to the final hours of the bacterial exponential growth phase. In addition, with an excess of propionate (70 mM), fluorescence was still observed.

Statistical analysis was performed to evaluate whether propionate concentration had a significant effect on fluorescence expressed by the bacteria. It was determined that the maximum propionate concentrations that induced normalized fluorescence were statistically different occurred between 5.5 and 7 h of growth. With the *post hoc* analysis, it was concluded that between these hours, there was sufficient evidence to say that propionate generated a statistically significant difference between 22 comparisons of normalized fluorescence expressed by the bacteria, with a significance of at least 5%. The results of the analysis for 6 h of growth are shown in Fig. 3c. The propionate biosensor between these hours was unable to express sfGFP levels that would allow differentiation between 50 and 70 mM propionate concentrations and between 20 and 30 mM and 30 and 40 mM concentrations of the compound.

**(ii) Butyrate biosensor.** Figure 4 shows the kinetics of both butyrate biosensors. As observed with the promoter test vector, an effect of butyrate concentration on the growth of both biosensors can also be seen, where the concentration increases and growth decreases. In this case, a greater effect was seen in butyrate biosensor K, because the high concentrations of butyrate generated a decrease in growth to almost half of the growth without the compound. In addition, at longer times, the growth curves for lower butyrate concentrations began to decrease, reaching a lower OD_600_ than that of the concentration curves that followed.

By analyzing the normalized fluorescence expression (Fig. 4b), the desired sensor behavior was achieved. Normalized fluorescence expression was inversely proportional to the butyrate concentration. Regarding the lower concentrations, at the beginning of the kinetics, butyrate biosensor K expressed practically equal normalized fluorescence for concentrations from 0 to 20 mM; therefore, it appears that the system is saturated at the maximum expression level. For biosensor C, although the 0 to 30 mM curves did not reach the same values, the difference between them was not statistically significant, as discussed later. As with the propionate biosensor, fluorescence expression was also observed with high concentrations of butyrate. It is noteworthy that butyrate biosensor K has a better difference in fluorescence expression levels for the different concentrations used than butyrate biosensor C. In addition, biosensor K showed higher fluorescence values at lower concentrations and lower values at higher concentrations. This may be related to the fact that the PpchA_*sfGFP_*K vector had higher protein expression and that when sfGFP was replaced with LacI, this was maintained and allowed better regulation of the reporter.

On the other hand, the same effect on growth was also observed. As time passed, the fluorescence curves grown at lower concentrations began to exhibit a lower growth rate, passing below the curves at higher concentrations. A decrease in the fluorescence of the lower concentration curves was even observed at the end of the kinetics and, inclusively, the 0 mM concentration curve reached a normalized fluorescence almost equal to the 60 mM butyrate curve at 40 h of kinetics in both systems (data not shown).

From the statistical analysis, it could be concluded that the maximum number of concentrations inducing statistically different normalized fluorescence means for butyrate biosensor C occurred between hours 17.5 and 21, and the test results at 21 h are shown in Fig. 4c. In these hours, there is sufficient evidence to suggest that butyrate has a significant effect on sfGFP expression in 19 comparisons, with a significance level of at least 5% (Fig. 4c). The values measured between these times do not allow us to conclude that concentrations of 0 to 30 mM butyrate cause sfGFP expression levels to differ from each other. The same was true for concentrations of 50 and 60 mM and 60 and 70 mM. For butyrate biosensor K, the maximum number of statistically different concentrations occurred between 11 and 17.5 h of growth. In this case, there were 24 comparisons with significant differences, and the calculated results for growth at 17.5 h are shown in Fig. 4d. At this time, almost all normalized fluorescence levels expressed by the bacteria were statistically different from each other, with at least 5% significance, except for the concentrations of 0 to 20 mM and 60 to 70 mM. Considering that the biological concentration of butyrate in the colon is between 10 and 20 mM (28) or up to 30 mM (12), the lower limits that both biosensors created in this study can detect may not allow differentiation of low butyrate concentrations in the biologically relevant range in IBD.

**(iii) Biosensor sensitivity.** Kinetics were then performed over a wider range of concentrations to determine the maximum and minimum concentrations to which the circuits remained sensitive, and the strength of the effect of SCFA on their regulation. For the propionate biosensor, growth was performed at concentrations between 0 and 130 mM propionate, including concentrations below 10 mM, to determine the lower limit of sensitivity. For the butyrate biosensor C, growth was performed at concentrations between 0 and 115 mM.

**FIG 5:**
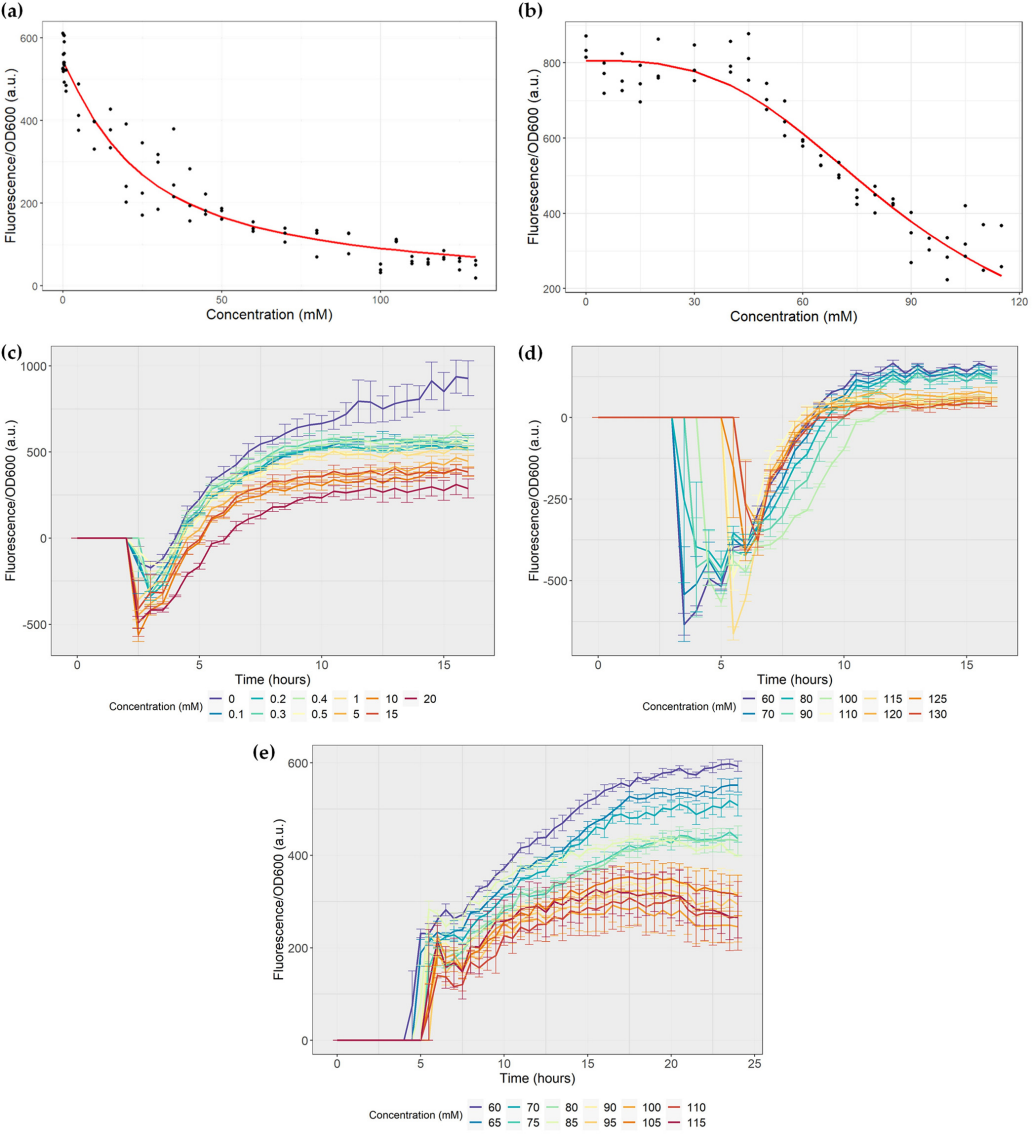
Biosensor sensitivity. The assays were performed in triplicate. a.u.: arbitrary units. (a) Hill equation fitting for the propionate biosensor at 15 h of growth. The objective function value of the parameter fit was 4.99, and the fitted parameters are as follows: β, 541.5 a.u.; *K*, 24.7 mM; and *n*, 1.15. (b) Hill equation fitting for butyrate biosensor at 20.5 h of growth. The objective function value of the parameter fit was 7.26, and the fitted parameters are as follows: β, 805.6 a.u.; *K*, 86.6 mM; and *n*, 3.14. (c) Normalized fluorescence of the propionate biosensor in the presence of 20 mM propionate. (d) Normalized fluorescence of the propionate biosensor growing between 60 to 130 mM propionate. (e) Normalized fluorescence of butyrate biosensor C growing between 60 to 150 mM propionate.

First, the Hill equation was adjusted for all hours of the study for both biosensors. The Hill function is used to model transcriptional regulatory networks describing the effect of the binding of transcription factors, considering the cooperative binding and ligand affinity for the receptor (29, 30). The adjusted parameters for the best five fits for each biosensor (that is, the five lowest objective functions) are detailed in Table 2, and the fit of the equation with the experimental data for the best fits is shown in Fig. 5a and b.

**TABLE 2:** Parameters of the five best fits of the Hill equation for each biosensor^*a*^

Biosensor	Hour	β (AU)	*K* (mM)	*n*	OF
Propionate	15	541.5	24.7	1.15	4.99
	16	547.5	26.1	1.18	4.99
	15.5	561.0	28.2	1.20	5.04
	14.5	550.6	27.1	1.17	5.14
	14	539.6	25.4	1.13	5.23

Butyrate	20.5	805.6	86.6	3.14	7.26
	21	818.8	84.3	3.10	7.27
	19.5	774.0	88.8	3.18	7.33
	18.5	748.7	89.6	3.18	7.45
	22	862.3	80.3	3.04	7.46

a β, maximum expression rate; *K*, dissociation constant; *n*, Hill cooperativity coefficient; OF, objective function.

As shown in Table 2, the best Hill cooperativity coefficient for the propionate system (n) is 1.15, with a 95% confidence interval of 0.98 to 1.34. Considering the five best fits, this coefficient ranges between 1.13 and 1.2. For the butyrate system, this coefficient has a value of 3.14, with a confidence interval of 2.69 to 3.64, and considering the five best fits, it ranges between 3.04 and 3.18. For the propionate biosensor, the value of the dissociation constant (*K*) is 24.7 mM, with a confidence interval of 20.7 to 28.7 in the best fit, and for the butyrate biosensor, *K* is 86.6 mM, with a confidence interval of 82.9 to 90.5.

Finally, to determine the limiting concentrations of SCFA that can affect sfGFP expression, the normalized fluorescence values for the highest and lowest concentrations tested are plotted in Fig. 5. A statistically significant difference with a *P* value less than or much less than 0.05 for fluorescence produced by the propionate biosensor was registered between 0 and 0.1 mM propionate after 12 h of growth (Fig. 5c), which suggests that the promoter P*_prpB_* is sensitive to very low concentrations of the inducer. However, no significant differences in sfGFP expression were observed between 0.1 and 1 mM and between 5 and 15 mM. Therefore, it is not possible to differentiate these concentrations from each other, but larger increments of propionate do appropriately affect the system. Regarding the upper limits (Fig. 5d), a slight difference was observed between the fluorescence produced in the presence of 60 to 80 mM propionate, but the 80 and 90 mM curves overlapped. From 110 mM propionate, the curves cross each other, making it impossible to differentiate the concentration levels and reaching the circuit’s saturation and the minimum expression point. It was confirmed that the groups of curves mentioned above were not statistically significantly different at a significance level of 5%. From the above analysis, it can be seen that the butyrate biosensor C was not sensitive to concentrations between 0 and 30 mM butyrate. As shown in Fig. 5e, the normalized fluorescence curves of concentrations 75 to 85 mM butyrate overlapped, and from a concentration of 90 mM, the curves also crossed each other, making it impossible to differentiate the concentration levels and reaching the minimum expression of the system or its maximum inhibition. It was confirmed that these curves were not statistically significantly different at a significance level of 5%, except for the comparisons of the curves for 75, 80, and 85 mM butyrate with that for 100 mM butyrate, which showed statistically significant differences from 14 h of growth.

### Biotherapeutics development.

Finally, we evaluated whether the designed circuit can also function as a biotherapeutic that expresses mouse granulocyte-macrophage colony-stimulating factor (mGM-CSF) without the presence of the corresponding SCFA and has minimal expression at high concentrations of the compound, so that it can express a cytokine that might counteract inflammation. GM-CSF is a 144-amino-acid (aa) peptide shown to improve clinical and histological parameters, reducing proinflammatory cytokines and improving intestinal mucosal repair in mouse models of dextran sulfate sodium (DSS)-induced acute colitis (31, 32). Furthermore, in a phase II clinical study, it was shown to decrease the severity and improve the quality of life of patients with moderate Crohn's disease (33). Three biotherapeutics were developed by cotransforming each sensor plasmid with the therapeutic plasmid Ptac_*mGMCSF*_SC101 (Table 1). The butyrate biotherapeutics were named butyrate biotherapeutics C and K based on the sensor plasmid used.

**FIG 6:**
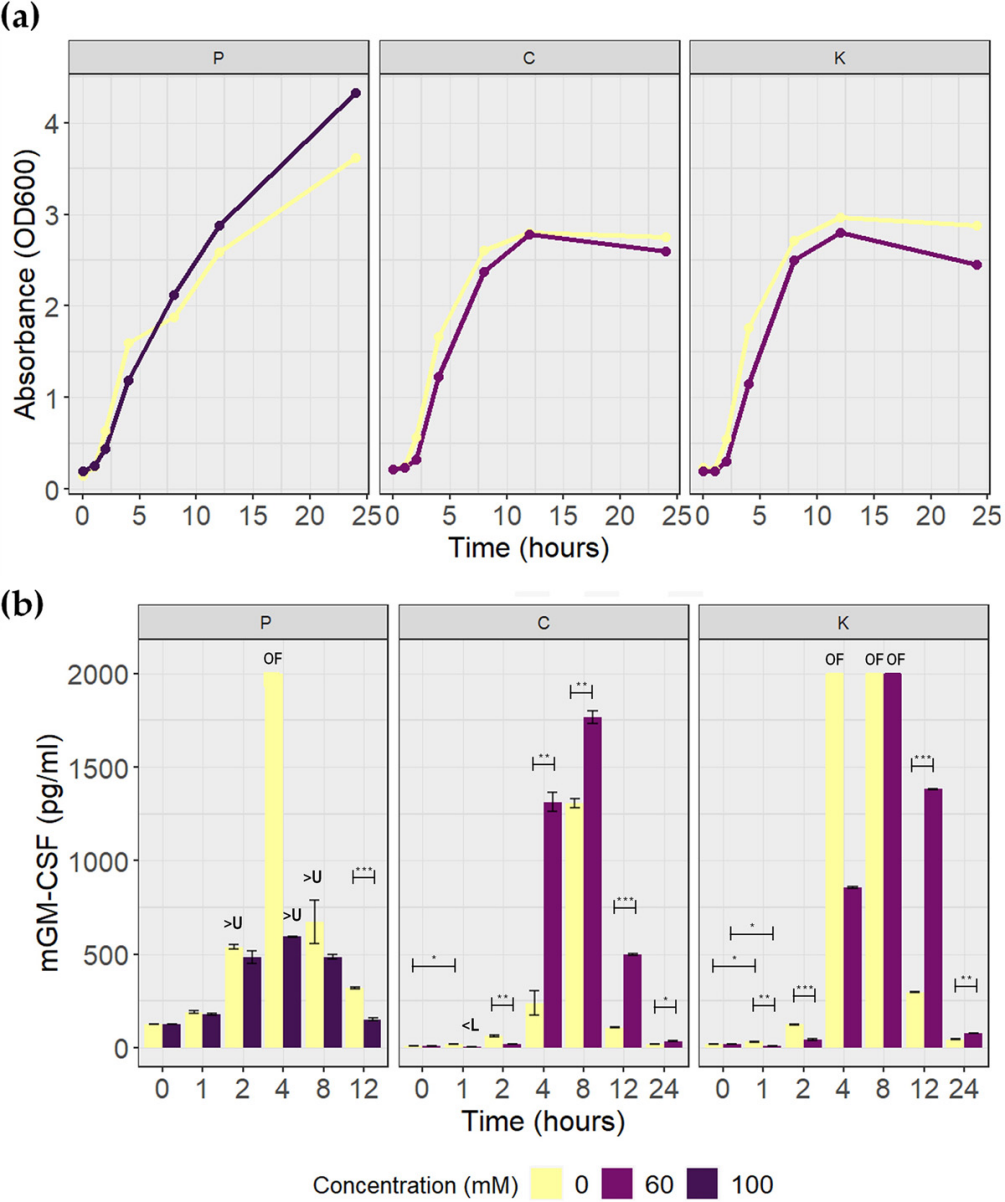
Quantification of mGM-CSF produced by biotherapeutics. P, propionate biotherapeutic; C, butyrate biotherapeutic C; K, butyrate biotherapeutic K. (a) Curves for growth in flasks. (b) Quantification of mGM-CSF expression by ELISA. The averages of two measurements and their standard errors are presented. The untransformed E. coli BL21 samples, with and without SCFA, were below the limit of quantification (3.9 pg/mL). OF, overflown, i.e., the protein concentration marked in these samples is not real, as it could not be calculated because it was outside the measuring range of the equipment. >U, higher than the upper limit of quantification, which in these cases was 500 pg/mL due to the dilution performed. <L, lower than the lower limit of quantification; in this case, no dilution was performed. Consider that the statistical tests were performed only at the time points where the concentrations of mGM-CSF produced were within the accurate measurement range under both conditions. *, *P* ≤ 0.05; **, *P* ≤ 0.01; ***, *P* ≤ 0.001.

Figure 6a shows that the propionate biotherapeutic did not reach the stationary growth phase within 24 h of culture under either of the two conditions. The growth in LB medium supplemented with 100 mM propionate started more slowly but reached higher absorbance levels, as observed in the growth of the biosensor. However, the growth dynamics of butyrate biotherapeutics C and K are very similar to each other. Even so, it was not possible to observe such a marked effect of butyrate concentration on bacterial growth, as observed in previous experiments.

Quantification of the mGM-CSF protein expressed under each condition by enzyme-linked immunosorbent assay (ELISA) is shown in Fig. 6b. The untransformed E. coli BL21 samples cultured with and without SCFA presented an absorbance below the limit of quantification of the test, which is equivalent to 3.9 pg/mL. This was expected, because this strain does not have the protein sequence and there is no protein expression. This result allowed us to conclude that the samples do not have any component that alters the quantification with the kit, and the values obtained correspond to the protein of interest. Some samples, even when diluted, presented very high protein concentrations, so the absorbance exceeded the limit measurable by the equipment and could not be quantified or showed absorbance above the upper limit of quantification of the kit; therefore, the values may not be accurate.

In the propionate biotherapeutic, more mGM-CSF was produced in the absence of propionate than in 100 mM propionate at each measurement point, which is in accordance with our objective (Fig. 6b). However, the difference between the two expression levels was smaller than expected, considering the large difference observed in sfGFP expression. Also, there was an increasing concentration of the cytokine in the presence of propionate, indicating that the circuit is not fully repressed.

For the butyrate biotherapeutics, both strains exhibit the expected behavior at the beginning of the kinetics, that is, lower protein expression in the presence of butyrate (Fig. 6b). During the first hour of growth, there was a decrease in the amount of cytokine expressed growing with butyrate, whereas growth without the compound showed an increase. Furthermore, in the case of butyrate biotherapeutic C, the concentration of mGM-CSF in the presence of butyrate was below the limit quantifiable by the test, and in the butyrate biotherapeutic K, the decrease in the amount from hour 0 to hour 1 of growth was statistically significant with a *P* value equal to 0.02. The increase in the amount of mGM-CSF in the absence of butyrate from hour 0 to hour 1 of growth for both biotherapeutics was also statistically significant. This shows that butyrate indeed inhibits protein expression by further inducing LacI, whereas in the absence of the compound, there was increased expression of mGM-CSF. Inhibition was still observed up to hour 2 for butyrate biotherapeutic C and up to at least hour 4 for butyrate biotherapeutic K. In the hours where the amount of mGM-CSF produced could be measured in both conditions, it was determined that the smaller amount of the cytokine produced in the presence of butyrate was statistically significant. At the following measurement points, a larger amount of mGM-CSF was observed in the presence of butyrate, contrary to what was expected. Butyrate biotherapeutic system K, however, showed an accelerated production of the cytokine at earlier times in the absence of butyrate than in its presence at 60 mM. This suggests that the ligand still represses mGM-CSF expression but that this effect is lost over time (Fig. 6b).

In addition, the dynamics of mGM-CSF expression is different from that of sfGFP. The concentration of mGM-CSF begins to increase until it reaches its peak at hour 4 in the propionate biotherapeutic and hour 8 in the butyrate biotherapeutics and then begins to decrease (Fig. 6b). Although this behavior cannot be concluded from the data for butyrate biotherapeutic K growing in the absence of butyrate, it is assumed that it must exhibit the same behavior as in the other three conditions. In the case of sfGFP, a decrease in fluorescence, which correlates with the amount of protein, was observed in the butyrate biosensors, but only for lower butyrate concentrations and at different times (Fig. 4b and data not shown).

## DISCUSSION

In this work, we designed and constructed gene circuits for sensing and releasing a protein in the presence of low concentrations of propionate and butyrate, essential SCFAs produced by the gut microbiota which participate in several physiological processes in the host.

We first used P*_prpB_*, a propionate-inducible promoter, for the development of the genetic circuits. In the regulation of P*_prpB_*, PrpR is a transcription factor responsible for promoter activation. The expression of the *prpR* gene, which is a σ^54^-dependent regulator (34), depends on the presence of the cyclic AMP (cAMP)-cAMP receptor protein (CRP) complex, and the transcription factor becomes activated in the presence of 2-methylcitrate (2-MC) (25, 35). 2-MC is produced from propionate via propionyl coenzyme A (propionyl-CoA) in the methyl citrate cycle (36). Therefore, the propionate-mediated activation of P*_prpB_* depends on the activated PrpR in addition to the cAMP-CRP complex itself, integration host factor (IHF), and σ^54^ and is directly mediated by 2-MC (25). The *prpR-*P*_prpB_* system was used to create a biosensor that was regulated over a wide range of propionate concentrations, and the production of the downstream protein varied directly and linearly with the extracellular propionate concentration (25). This system was found to be slightly “leaky”; background expression of *gfp* was detected in the absence of an exogenous inducer (25).

P*_pchA_* is a butyrate-induced promoter. It was shown to play a fundamental role in the butyrate-mediated induction of LEE1 genes, a process directly mediated by the PchA protein (26). Upon *pchA* deletion, butyrate-mediated induction of LEE1 protein expression was lost, as was the butyrate-induced activity of the LEE1 operon promoter. The induction of LEE1 operon promoter activity was recovered when *pchA* was incorporated into a plasmid with its native promoter, as opposed to the incorporation of the gene controlled by the P*_tac_* promoter (26). Despite this, it is not known which factor mediates the activation of the *pchA* gene by butyrate and which is the sequence of the regulatory site or the operator that is activated by the compound in the P*_pchA_* promoter. Therefore, we decided to use the unnarrowed sequence upstream of the gene used by Nakanishi et al. (26), where butyrate-mediated regulation was observed but without including the coding sequence of the gene.

Although it has been reported that E. coli can use propionate as its sole carbon source (37), we observed low overnight growth in minimal medium supplemented with a wide range of propionate concentrations. We hypothesize that because the bacteria take a prolonged amount of time to adapt to this medium and because SCFA was being used as a carbon source to generate biomass, it was not being used to induce the genetic system that we developed. Therefore, to ensure adequate bacterial growth and the use of propionate to activate the genetic circuit, we decided to grow our strain in LB medium, which is nutrient rich. It was observed that both the biosensor and the biotherapeutic achieved higher growth in the presence of propionate than in its absence (Fig. 3a and 6a). These results indicate that, although they were in LB medium, it is likely that bacteria additionally used propionate as a carbon source to generate biomass. In addition, the duration of the lag phase generally increased with propionate concentration, indicating that bacteria took longer to adapt to the medium when it was enriched with propionate. Although the concentrations of propionate used in this study to supplement LB medium had a positive impact on bacterial growth, it has previously been reported that very high concentrations of the SCFA can have an inhibitory effect on growth (38). This is thought to be caused by the accumulation of 2-MC, which may block fructose-1,6-biphosphatase, a key enzyme in the gluconeogenesis pathway (38), and can explain the increasing duration of the lag phase with increasing concentrations of propionate observed in this study.

Throughout the study, it was observed that systems in which the P*_pchA_* promoter was cloned into the plasmid containing kanamycin as a resistance marker, PpchA_*sfGFP*_K and PpchA_*lacI*_K (Table 1), expressed more protein (Fig. 1b, 4b, and 6b). This observation is in agreement with that reported by Iverson et al. (39). They evaluated expression levels using the same transcriptional unit but in vectors with different antibiotic resistance genes. Most of the transcriptional units assembled in the vector with kanamycin resistance expressed more protein than their equivalent units in ampicillin resistance vectors. The mechanisms of action of kanamycin and carbenicillin differ from each other. Kanamycin is an aminoglycoside bactericidal antibiotic that binds to the bacterial 30S ribosomal subunit, causing interference with the initiation complex and misreading of tRNA, leaving the bacteria unable to synthesize proteins that are vital to their growth (40). The product of the resistance gene *kanR*, also called kanamycin kinase or aminoglycoside 3′-phosphotransferase, inactivates kanamycin by phosphorylating it (41). Carbenicillin is a bactericidal β-lactam antibiotic that inhibits cell wall biosynthesis by binding to one or more penicillin-binding proteins, thereby inhibiting the final transpeptidation step of peptidoglycan synthesis (42). The *bla* (β-lactamase) resistance gene encodes an enzyme that inactivates β-lactam antibiotics by splitting the amide bond of the β-lactam ring (43). Both antibiotic resistance genes act by inactivating antibiotics, but β-lactamase is secreted and inactivation occurs extracellularly, while kanamycin inactivation occurs intracellularly because the target of the antibiotic is cytoplasmic (44). Since kanamycin acts by affecting the production of proteins, causing the expression of nonfunctional or toxic proteins (40), it is expected that when this antibiotic is used, the production of plasmid proteins would decrease. However, the results show the opposite trend.

High basal expression of the P*_pchA_* promoter was observed and could explain the kinetics of sfGFP and mGM-CSF. This could be due to the characteristics of the promoter sequence that allow the binding of the transcriptional machinery even though the activator is not present, as is the case, for example, when the P*_tac_* promoter is active even when the repressor is present (45, 46). Another reason could be that unwanted regulatory sites may be included in the selected promoter sequence. Since the regulatory region of the compound of interest and, moreover, how the regulation itself occurs are not yet known, an unbounded 500-bp sequence is being used as the promoter region. It is likely that there are binding sites for other regulators that activate transcription and alter its regulation. An attempt could be made to narrow down the promoter and identify the operator region(s), thus decreasing the likelihood of other metabolites regulating the promoter. For this, one option would be to design test plasmids with different regions of the promoter upstream of the reporter protein and identify the regions in which regulation is not lost and/or has a more desired behavior. With this methodology, it was possible to identify a sequence of the LEE promoter that was upregulated by P*_pchA_* (47), as an example.

Fluorescence was also observed in the three biosensors with high concentrations of the corresponding SCFA. This could be because the P*_tac_* promoter is very leaky; that is, it has a high basal expression (46). Even if the repressor is present, there is still protein expression downstream of the P*_tac_* promoter that generates protein accumulation, even in a low-copy-number vector (45). Therefore, even if high concentrations of the SCFA are added, sfGFP expression will always occur, because transcription cannot be completely repressed by the P*_tac_* promoter. Another reason for the expression with high butyrate concentrations could be that the P*_pchA_* promoter could be characterized as a strong promoter, and as this transcriptional unit is in a vector with a high-copy-number origin site, this combination would cause a very high metabolic load that would trigger a decrease in LacI production, as seen in other studies (46, 48). The option of changing the pTAC promoter to one that is more sensitive to LacI is proposed to obtain a more precise and controlled response of the circuit. This yielded very good results in a study by Wang et al. (45). Another option would be to change the promoter-repressor pair to another pair with better regulation and more sensitivity.

It was observed that over time, the fluorescence curves of the butyrate biosensors at lower concentrations began to exhibit a lower expression rate, passing below the curves at higher concentrations (Fig. 4b). Even the 0 mM concentration curve reached a normalized fluorescence almost equal to that of the 60 mM butyrate curve at 40 h of kinetics in both systems. This behavior could be due to the possibility that there are undetected regulation sites for metabolites that appear later in kinetics or accumulate over time. These sites can generate regulations that are not considered and that affect the behavior and logic of the designed circuit. At low butyrate concentrations, large amounts of LacI should not be induced, as seen with the promoter test vectors (Fig. 1b), but there may be an activator-binding site that could lead to an induction of LacI over time, which decreases sfGFP expression. The same effect, but more marked, was observed in butyrate biotherapeutics, where even the logic of protein production was inverse to what was expected after a period of time. This could be because late in the kinetics, the rate of mGM-CSF expression may begin to decrease in LB without butyrate, or a higher expression of mGM-CSF, perhaps due to lower inhibition, begins to be induced in the presence of the compound. Considering that the propionate biotherapeutic circuit works correctly, and the only difference between the propionate and butyrate systems is the promoter used, the hypothesis that butyrate biotherapeutics no longer exhibit the desired behavior over time due to the unbound P*_pchA_* promoter, unlike the P*_prpB_* promoter, is further supported.

The expression, accumulation, and duration of an overexpressed heterologous protein are affected by several factors, such as the strength of the promoter and ribosome binding site, the amount of inducer, the induction time, the recombinant protein's own characteristics (such as size, solubility, or other physical and biochemical characteristics), degradation times, response of the cell to protein expression, and interaction with the biological processes of the bacterium, among many others (49). The difference in the expression dynamics of the circuit when changing the response protein may be mainly due to the characteristics of the proteins and perhaps bacterial growth, which may affect the metabolic state in which they are found.

Regarding the sensitivity and affinity of the circuits to SCFAs, since the Hill cooperativity coefficient in both systems is greater than 1 (Table 2), it is said that there is positive cooperativity in the regulation of both circuits (43); for the butyrate biosensor, which is well over 1, it can be said that there is high cooperativity in that system. Although the value of *n* is often associated with the number of molecules that must be joined to produce the desired effect, it is not entirely correct (30). The Hill coefficient incorporates several effects (51). It considers the affinity and efficiency of the regulatory factors to bind and control the circuit. In addition, it incorporates the binding energy and determines whether the binding of more factors is required to achieve regulation. If one analyzes a single regulation, a positive cooperativity could imply that there is a need for more than one SCFA molecule for the regulation of the system, that the transcription factor in charge of regulation has two or more subunits that must bind to be functional, or that the binding of one molecule facilitates the binding of more molecules for regulation (29, 51, 52). However, as the circuit analyzed is a gene-regulatory network consisting of two regulations, the effects of SCFA and LacI regulation are being added, so the interpretation of the value of *n* becomes more complex (29). Moreover, it must be considered that, in the case of the butyrate system, it is unknown which is the direct transcription factor of the system and how it is regulated, so it is even more complex to interpret.

The dissociation constant represents the equilibrium between bound and unbound SCFA in the system. This corresponds to the concentration of SCFA required to achieve 50% repression (51). Moreover, it is inversely related to the apparent affinity between the regulator and the designed circuit, and low values imply high affinity for the regulator (52). The *K* value of the propionate biosensor, 24.7 mM, is interesting because the concentration of propionate in fecal samples from healthy patients is approximately 20 mM (9), which is likely higher in the colon. This indicates that sfGFP production is considerably low in the presence of concentrations of propionate which may be a sign of healthy gut conditions. Conversely, for the butyrate biosensor, the high *K* value of 86.6 mM, implies that the circuit does not have a high affinity for butyrate and that it requires a high concentration, much higher than the biological concentration in humans, to repress 50% of the maximum sfGFP expression. Considering the biological concentrations in the gut, the system would be turned on at a high level most of the time. This, in addition to the fact that the lower limits that the butyrate biosensors can detect may not allow differentiation of low butyrate concentrations in the biologically relevant range in IBD, shows the need to make adjustments to the designed circuit in order for it to have real applications for human health. This can be achieved, for example, by using synthetic biology tools to achieve a more suitable behavior for the desired application of the system. It is possible to fine tune the expression levels of each of the transcriptional units by testing and interchanging different biological parts, such as ribosome binding sites with different affinities, origins of replication, and promoters and terminators of various strengths, and by incorporating translational regulation sites or sequences that modify the stability of mRNA or proteins, among many options (45, 51).

Finally, we consider that this study serves as a proof of concept, and further developments are needed. As the aim of developing these biosensors and biotherapeutics is to use them as diagnostic and therapeutic tools in a biological context, the genetic circuits must be integrated into the genome in the final prototype. This will reduce the possibility of possible mutations, the loss of part of the circuit, and the use of antibiotic resistance genes as a selective pressure. Fine-tuning by adjusting ribosome binding site (RBS) and promoter sequences could also modify the dynamic range of detection. In addition, the means for GM-CSF secretion must be determined. The simplest solution may be to use the synchronized lysis circuit integrated into the genome of E. coli Nissle 1917, developed by Din et al. (53) and Gurbatri et al. (54), in which the bacteria lyse when reaching a certain density. This system has been used to release therapeutic substances for cancer treatment. Last, the system should be tested *in situ* and *in vivo* to determine how a more realistic environment may interfere or possibly improve the dynamics of the genetic circuit and analyze the effects of GM-CSF on the intestinal epithelium as a biotherapeutic produced *in situ*.

### Conclusions.

In conclusion, we have created propionate-repressible and butyrate-repressible expression systems that allow the production of increasing amounts of a desired protein in the presence of decreasing propionate or butyrate concentrations. We observed that the propionate biosensor functioned within a range of approximately 0 to 110 mM SCFA, and the butyrate biosensor functioned within a range of 30 to 90 mM SCFA. The propionate biotherapeutic expressed larger amounts of mGM-CSF in the absence of propionate at each measurement point, and the butyrate biotherapeutics presented the expected behavior only at the beginning of kinetics. Currently, the biosensors may function as diagnostic tools for quantifying propionate or butyrate levels. For the biotherapeutics, further studies should be performed to optimize the conditions for the differential expression of the cytokine in the presence of various concentrations of SCFA. These results show promise, although further fine tuning of the circuit response ranges and detection limits is required to improve the sensing and response mechanisms. In order to evaluate the potential of engineered bacteria as a tool to sense and respond to SCFA levels in the intestine and improve mucosal barrier integrity, *in vivo* studies should also be carried out.

## MATERIALS AND METHODS

### Bacterial strains and plasmids.

E. coli DH5α was used for the propionate biosensor, and E. coli BL21 was used for the butyrate biosensor and both biotherapeutics. The P*_pchA_* promoter was amplified from the E. coli ATCC 35150 serotype O157:H7 genome (referred to here as E. coli O157:H7), which was kindly donated by Magaly Toro from Universidad de Chile, while the *lacI* gene repressor was amplified from the E. coli K-12 MG1655 genome (referred to here as E. coli K-12).

The plasmids and primers used in this study are listed in Tables 1 and 3, respectively. As the plasmids were assembled using the Gibson Assembly technique (55), all primers were designed to amplify the parts to be assembled and the backbone vectors, adding complementary overhangs to the other sequence. All PCR products were run on agarose gels and purified using the Zymoclean gel DNA recovery kit (Zymo Research, USA). The backbone and insert of each plasmid assembly were added at a ratio of 1:3 to Gibson Assembly master mix (New England Biolabs, USA). The mixture was then incubated at 50°C for 1 h.

**TABLE 3:** Primers used in this study^*a*^

Purpose and product	Template	Sequence (5′→3′)
PprpB_*lac*I assembly		
*lacI*	E. coli K-12 genome	AAGCTAGCAGGAGGAATTCAgtgaaaccagtaacgttatacgatg
		CCAAGCTTGCATGCCTGCAGtcactgcccgctttccagtc
Backbone	pPro24	gactggaaagcgggcagtgaCTGCAGGCATGCAAGCTTGG
		tataacgttactggtttcacTGAATTCCTCCTGCTAGCTTGT
PpchA_*sfGFP_*C and PpchA_*sfGFP_*K assembly		
P*_pchA_*	E. coli O157:H7 genome	CAGTCACGACGTTGTAAAACcacaggaatatatccgtaccc
		AGTTCTTCTCCTTTGCTCATaagtgacctccgattatctac
Backbone	Ptac_*sfGFP_*ColE1_C/Ptac_*sfGFP_*ColE1_K	TAGATAATCGGAGGTCACTtatgagcaaaggagaagaact
		GGTACGGATATATTCCTgtGgttttacaacgtcgtgactg
PpchA_*lacI*_C and PpchA_*lacI*_K assembly		
*lacI*	E. coli K-12 genome	TCTGTAGATAATCGGAGGTCACTtgtgaaaccagtaacgttatacgatgtcgc
		GACAGGTTTCCCGActaactcacattaattgcgtt
Backbone	PpchA_*sfGFP_*C/PpchA_*sfGFP_*K	CGCAACGCAATTAATGTGAGTTagtcgggaaacctgtcgtg
		ACGTTACTGGTTTCACaagtgacctccgattatctacagactgccatcc
Ptac_*mGMCSF*_SC101 assembly		
mGM-CSF gene	pPro_*mGMCSF*	cacacaggaaacagaattctATGGCGCCGACACGCTCTCC
		tagatcagctaattaagcttCTATTTCTGTCCAGGCTTTTTGCACTCG
Backbone	Ptac_*sfGFP_*SC101	AAAAGCCTGGACAGAAATAGaagcttaattagctgatctagacg
		GGAGAGCGTGTCGGCGCCATagaattctgtttcctgtgtga

a The lowercase sequences are complementary to the template, and the uppercase sequences correspond to the overhangs required for the Gibson Assembly technique. The first primer in each pair is the forward primer.

For the propionate sensor plasmid, *lacI* was cloned into the pPro24 backbone, including the *prpR* sequence, to replace GFPuv. For the butyrate sensor plasmid, the first promoter test vectors were created by replacing the P*_tac_* promoter from Ptac_*sfGFP*_ColE1_C or Ptac_*sfGFP_*ColE1_K with the P*_pchA_* promoter. The *lacI* gene was then cloned, replacing the sfGFP gene, in these promoter test vectors. Finally, to create the therapeutic response plasmid Ptac_*mGMCSF*_SC101, the mGM-CSF gene was amplified from pPro_*mGMCSF* and cloned to replace the sfGFP gene in Ptac_*sfGFP_*SC101. The correct insertion and sequences of the P*_pchA_* promoter in the promoter test vectors, *lacI* in the propionate biosensor, and the mGM-CSF gene in therapeutic response plasmids were verified through plasmid sequencing at Macrogen, Inc.

### Bacterial transformation.

The vectors assembled were transformed into chemically competent E. coli DH5α. Two microliters of a 1:4 dilution of the assembly reaction mixture was added to 100 μL of bacteria. The bacteria were then incubated on ice for 30 min and heat shocked at 42°C for 50 s, followed by incubation for 2 min on ice. Then, 900 μL of super optimal broth Super Optimal broth with Catabolite repression medium was added, and the bacteria were incubated at 37°C with shaking at 250 rpm for 1 h. The entire transformation volume was plated on LB agar medium with the corresponding antibiotic(s). Finally, isolated colonies were randomly selected for growth in LB medium containing the corresponding antibiotic(s) to be tested and stored. The same protocol was used for chemically competent E. coli BL21 after plasmid purification using miniprep with an E.Z.N.A. plasmid DNA minikit II (Omega Bio-tek, USA), following the manufacturer’s protocol. For the cotransformations, 1.5 μL of each plasmid was added to the bacteria, and the incubation was 2 h instead of 1 h. After each transformation and at different stages of the study, colony PCRs were performed to confirm that the sequences of the promoter, the sfGFP gene, *lacI*, and/or the mGM-CSF gene were present in the strain used, as appropriate. All clones were cultured under the selective pressure of the antibiotic(s) to which they are resistant thanks to the plasmid(s) they possess, to ensure that none of the transcriptional units had been lost.

### Kinetics of sfGFP expression.

To evaluate P*_pchA_* promoter activation dynamics and characterize the behavior of the biosensors, 16-, 24-, or 40-h kinetics were performed in black 96-well plates with transparent bottoms. Kinetics were performed using a Synergy H1 plate reader (BioTek, Agilent Technologies) at 37°C with constant double orbital shaking. Absorbance at 600 nm and fluorescence with excitation and emission wavelengths of 485 and 510 nm, respectively (50), were measured every 30 min to characterize the growth and sfGFP expression of the different strains created.

Bacteria were first grown overnight with the corresponding antibiotic(s) at 37°C, with shaking at 250 rpm. The next day, they were inoculated at a final concentration of 1% in fresh LB with antibiotics and supplemented with sodium propionate to achieve concentrations between 0 and 130 mM or sodium butyrate to achieve concentrations between 0 and 115 mM in the different experiments. 200 μL was added to each well, with duplicates or triplicates of each concentration tested depending on the experiment.

To analyze the results, we first subtracted the average fluorescence and OD_600_ of the wells with medium without bacteria from all the wells at each measurement time point. As larger amounts of biomass would produce more sfGFP, we decided to analyze the normalized fluorescence by dividing the measured fluorescence values by the OD_600_ at each measurement time. For OD_600_ values less than 0.09, the normalized fluorescence values in several cases were extremely large because of the very small denominator, which provides values that do not make biological sense. Therefore, these values were set to zero to better plot the results of all analyses. Finally, the averages and standard deviations of the data were calculated.

Analysis of variance (ANOVA) and Tukey’s test were used to determine whether the SCFA concentrations had a significative effect on normalized fluorescence expressed by the biosensors. The test was done for each measurement time point.

Finally, to determine the sensitivity of the circuit to the corresponding SCFA, the Hill equation for a repressor was fitted to the normalized fluorescence versus butyrate concentration for each measurement. The following equation was used (51):

$$\frac{d\;(\text{F}/\text{OD})}{dt}=\frac{\beta K^{n}}{K^{n}+{\left[s\right]}^{n}}$$

where F/OD corresponds to the normalized fluorescence, β is the maximum expression rate, *K* is the dissociation constant, [*s*] is the SCFA concentration, and *n* is the Hill cooperativity coefficient.

Parameter estimation was performed by minimizing the following objective function (OF):

$$\text{OF}=\frac{\sqrt{\sum \;{\left(\text{F}/{\text{OD}}_{\text{experimental}}\;-\;\text{F}/{\text{OD}}_{\text{calculated}}\right)}^{2}}}{\text{number of data points}}$$

where the numbers of data points used were 81 and 63 for the propionate and butyrate biosensor fitting, respectively. Confidence intervals were then calculated for each parameter obtained in the best fit for each biosensor using the confint function of R.

### Kinetics of mGM-CSF expression.

Both butyrate biotherapeutics (that is, bacteria transformed with a butyrate sensor plasmid and the therapeutic plasmid with mGM-CSF) and the propionate biotherapeutic (a bacterial strain transformed with the propionate sensor plasmid and the therapeutic plasmid) were cultured overnight with the corresponding antibiotics. The next day, 1-L flasks with 300 mL LB or LB with the corresponding SCFA with antibiotics were inoculated at an initial OD_600_ equal to 0.2 for the three bacteria. Two flasks, LB and LB with 100 mM sodium propionate, were used for the propionate biotherapeutic, and two flasks, LB and LB with 60 mM sodium butyrate, were also used for each of the butyrate biotherapeutics, for a total of four butyrate biotherapeutic cultures.

Samples were collected at 0, 1, 2, 4, 8, 12, and 24 h. At each time point, 1 mL was collected to quantify the OD, and 5 mL was used to extract the total proteins from each culture. The amount of bacteria used for protein extraction was normalized from the lowest OD_600_ for butyrate and propionate biotherapeutics. For each sampling time, the volume used for the extraction of each culture was calculated using the following equation:

$${\text{volume}}_{\text{culture}}=\frac{\text{5 ml}\times {\text{OD}}_{600_{\text{minor}}}}{{\text{OD}}_{600_{\text{culture}}}}$$

where ${\text{OD}}_{{\text{600}}_{\text{minor}}}$ corresponds to the lowest OD_600_ measured among four or two cultures at each sampling time.

The same procedure was followed for a negative control using an untransformed BL21 culture grown with 60 mM butyrate, 100 mM propionate, and no SCFA but sampled only at 8 h, the time of maximum mGM-CSF expression, in a preliminary experiment. The number of bacteria was normalized based on the lowest OD_600_ measured for the biotherapeutics at that time.

### Protein extraction and quantification.

Protein extraction for butyrate biotherapeutics was based on the protocol described by Mikami et al. (56). Standardized samples were centrifuged at 4,000 rpm for 15 min at 4°C. The pellet was resuspended in 500 μL of wash buffer (20 mM Tris HCl and 0.15 M NaCl, pH adjusted to 7.5) and centrifuged again under the same conditions. The supernatant was discarded, and the pellets were frozen in liquid nitrogen and stored at −20°C. The pellets were then resuspended in 350 μL of lysis buffer (0.1 M KCl, 20 mM HEPES, 10% glycerol, 0.1% Triton X-100, 5 mM 2-mercaptoethanol, EDTA protease inhibitor). Bacteria were lysed by sonication on a model 250 digital Sonifier (Branson), using the conditions recommended by the supplier. Samples were subjected to 6 cycles of 20 s on/10 s off at 30% amplitude on ice. They were then centrifuged at 20,000 × *g* for 30 min at 4°C, and the supernatant was stored at −80°C until use.

For the propionate biotherapeutic, the standardized samples were centrifuged under the conditions described above. The supernatant was discarded, and the pellets were stored at −20°C. The pellets were resuspended in 60 μL of Bugbuster protein extraction reagent (Millipore, USA), to which 3 μL of lysozyme at 50 mg/mL was added. The lysed bacteria were centrifuged for 15 min at 4°C, after which the supernatant was stored at −80°C until use. For protein quantification, the samples were thawed on ice and centrifuged at 16,000 × *g* for 15 min to sediment possible protein aggregates that could affect the measurement.

The mouse GM-CSF ELISA kit (Invitrogen Thermo Fisher Scientific, USA.) was used to quantify GM-CSF production under each condition. Each time point was quantified in duplicate following the manufacturer’s protocol. However, for the butyrate biotherapeutic samples, instead of using a 1:2 dilution of the samples, either the complete sample was added or a 1:10 dilution of the samples that were out of the quantification range in a preliminary assay was used. These diluted samples were from 4, 8, and 12 h. The propionate biotherapeutic samples were quantified only up to hour 12.

ANOVA was used to evaluate whether the presence of SCFA had a significant effect on the amount of mGM-CSF expressed by the biotherapeutics. The test was performed at each measurement time point where the amount of mGM-CSF produced could be correctly measured under both conditions, with and without SCFA, for each biotherapeutic. In addition, in the butyrate biotherapeutics, ANOVA was also performed to evaluate whether the decrease or increase in the amount of cytokine expressed in the first hour of growth was statistically significant.

### Data availability.

Raw data from this work are presented in the “raw data el” Excel file (https://github.com/ksd-1/SCFA-Repressed-Biotherapeutic-Production), which contains fluorescence and OD results of each experiment as well as ELISA results.
